# Gene Expression Dynamics Accompanying the Sponge Thermal Stress Response

**DOI:** 10.1371/journal.pone.0165368

**Published:** 2016-10-27

**Authors:** Christine Guzman, Cecilia Conaco

**Affiliations:** Marine Science Institute, College of Science, University of the Philippines, Diliman, Quezon City, Philippines; University of Connecticut, UNITED STATES

## Abstract

Marine sponges are important members of coral reef ecosystems. Thus, their responses to changes in ocean chemistry and environmental conditions, particularly to higher seawater temperatures, will have potential impacts on the future of these reefs. To better understand the sponge thermal stress response, we investigated gene expression dynamics in the shallow water sponge, *Haliclona tubifera* (order Haplosclerida, class Demospongiae), subjected to elevated temperature. Using high-throughput transcriptome sequencing, we show that these conditions result in the activation of various processes that interact to maintain cellular homeostasis. Short-term thermal stress resulted in the induction of heat shock proteins, antioxidants, and genes involved in signal transduction and innate immunity pathways. Prolonged exposure to thermal stress affected the expression of genes involved in cellular damage repair, apoptosis, signaling and transcription. Interestingly, exposure to sublethal temperatures may improve the ability of the sponge to mitigate cellular damage under more extreme stress conditions. These insights into the potential mechanisms of adaptation and resilience of sponges contribute to a better understanding of sponge conservation status and the prediction of ecosystem trajectories under future climate conditions.

## Introduction

Global mean temperature will continue to rise over the 21^st^ century with the best estimates for global sea surface temperature (SST) increasing in the range of 1–3°C [1]. This increased SST may have deleterious impacts on many marine invertebrates. In fact, mass coral bleaching events triggered by elevated seawater temperatures have resulted in significantly reduced coral cover throughout the tropics [2]. This decrease in coral cover can result in changes in benthic reef communities [3], which may allow other species, such as algae and sponges, to increase in abundance. Sponges are one of the earliest multicellular animal groups [4]. Members of this phylum display remarkable ecological adaptability, having integrated into diverse marine ecosystems through the development of complex physiological and chemical properties [5]. Sponges play important roles in the functioning of these ecosystems [6].

Despite the diversity within the sponge phylum, data on sponge conservation status is still lacking [7]. Out of the thousands of known sponge species, very few are currently listed as threatened, suggesting that sponges are highly adaptable to environmental stressors, such as elevated temperatures, ocean acidification, sedimentation, and microbial pathogens [7]. In fact, compared to other reef taxa, marine sponges have the potential to be resilient to large-scale thermal stress events. Recent studies have reported that sponges are more tolerant to increased SST, with larval dynamics, ecological functions and physiological processes unaffected by increases in water temperature [8–10]. For example, the growth and survival of several Carribean sponges remained unaffected by exposure to thermal stress [11]. Furthermore, the sponge assemblage in Bahia, Brazil, did not change between pre- and post-El Niño Southern Oscillation (ENSO) years [12]. In contrast, some studies have described the negative effects of elevated temperature on different sponge species. For example, adult colonies of *R*. *odorabile* were found to be highly sensitive to thermal stress at 32°C [9] while severe sponge die-off due to cyanobacterial decay was triggered by elevated temperatures in the Mediterranean sea [13]. Therefore, it is important to recognize that different sponge species may have variable responses to environmental perturbations, specifically to thermal stress.

The molecular mechanisms underlying sponge responses to thermal stress are poorly understood, with most studies focusing on the effect of temperature on the sponge-associated microbial community. In one example, 454 pyrosequencing of the 16S rRNA metagenome revealed that exposure to 31°C had no effect on the bacterial biosphere within the Great Barrier sponge *Rhopaloeides odorabile* [14]. Likewise, in the Mediterranean Sea sponge *Ircinia spp*., neither thermal stress combined with food shortage nor large fluctuations in temperature and irradiance disrupted the stability of the sponge-bacteria partnership [15,16]. The importance of cell-cell signaling genes in the maintenance or breakdown of the sponge-bacteria interaction during thermal stress events has been explored to some extent [17]. Targeted studies on gene expression have demonstrated the upregulation of heat shock protein 70 in the Carribean sponge *Xestospongia muta* upon exposure to elevated temperature [18]. Expression profiling by multiplexed reverse-transcription quantitative PCR (mRT-qPCR) showed that *R*. *odorabile* larvae are remarkably able to withstand seawater temperatures up to 9°C above normal [9]. The apparent resilience of some sponge species and the sensitivity of others highlights the need to understand the genomic basis of sponge responses to environmental stressors and how they are able to adapt to rapidly changing ocean conditions.

High-throughput transcriptome sequencing is a powerful tool that allows sensitive and high-resolution detection of a wider dynamic range of expression levels in contrast to other commonly used molecular approaches, such as quantitative PCR, multiplex reverse-transcription quantitative PCR, and microarrays [19]. Global transcriptome analysis can reveal how organisms respond to external stimuli and stressors by detecting changes in gene expression dynamics. This plasticity of gene expression underlies the ability of organisms to adapt to changing environmental conditions. In fact, sequencing of the genome of model demosponge, *Amphimedon queenslandica*, revealed the presence of a diverse genetic toolkit, or a set of conserved regulatory genes, that can integrate various signaling pathways and allow the organism to rapidly respond to its environment [20].

In this study, we performed a transcriptome-wide analysis to elucidate the changes in gene expression that occur when a sponge is subjected to different levels of thermal stress. In particular, we focused on the differential expression of genes involved in protein folding, oxidative stress response, immune response, signal transduction, transcriptional regulation, apoptosis, and tissue morphogenesis. The findings of this study unveil key processes that underlie sponge tolerance to thermal stress, which will gain importance as ocean temperatures continue to rise with the changing climate.

## Results and Discussion

As global warming continues to raise ocean temperatures, marine ecosystems are placed at risk. However, some marine organisms, including sponges (Porifera), thrive in naturally warm environments and can tolerate high temperatures. Studies on these organisms will contribute to our understanding of the mechanisms that confer resilience to thermal stress. Thus, to elucidate the transcriptome dynamics underlying the sponge thermal stress response, we focused our study on the demosponge, *Haliclona tubifera*, found native to shallow reef flats in Bolinao, Pangasinan, Philippines. Based on regular monitoring by the Bolinao Marine Laboratory, sea surface temperatures in this region range from 25°C to 32°C with an annual mean temperature of 28.89±0.90°C. Sponges in this area are of great interest in exploring the determinants of ecological success, particularly in terms of tolerance to a wide range of temperatures.

**Fig 1:**
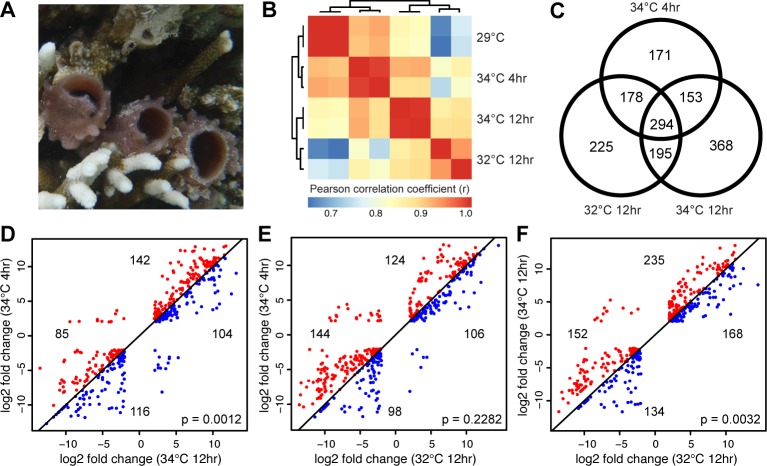
Global transcriptome profile of adult colonies of *H*. *tubifera* exposed to thermal stress. (A) *H*. *tubifera* is a soft, pink or brownish, tubular sponge found in association with coral skeletons in shallow water reef flats. (B) Correlation of overall gene expression profiles for duplicate samples of sponges exposed to different thermal regimes (Pearson correlation coefficient, r). The correlation is based on counts per million (CPM) of reads mapping to each transcript. Only transcripts with CPM >10 in at least 2 samples were included. (C) The number of differentially expressed genes specific to or common between different treatments. Differential expression analysis was conducted on duplicate samples for each experimental treatment. Genes were considered differentially expressed if they were up or downregulated by greater than 4-fold relative to the controls with an adjusted p-value <1x10^-5^. Scatter plots of the log2 fold changes in expression relative to controls at 29°C for differentially expressed genes that are common between (D) 34°C 4hr vs 34°C 12hr, (E) 34°C 4hr vs 32°C 12hr, and (F) 34°C 12hr vs 32°C 12hr samples. Points in the upper right quadrant are upregulated transcripts while points in the lower left quadrant are downregulated transcripts. Red dots above the diagonal represent transcripts with a greater magnitude of change in the sample on the y-axis while blue dots are transcripts exhibiting a greater magnitude of change in the sample on the x-axis. Enrichment of genes with particular distributions between treatment pairs was estimated using Fisher’s exact test (p-values shown).

*H*. *tubifera* belongs to order Haplosclerida, which is the largest and most diverse group within class Demospongiae of phylum Porifera (sponges). This tubular pink sponge is found in association with branching corals in shallow water reef flats at depths of 1–2 meters (Fig 1A). Because of its shallow habitat, this sponge species is subjected to widely fluctuating temperatures on a daily basis. The reference transcriptome used in this study was previously assembled *de novo*, and contains 50,067 non-redundant transcripts, which translate into 18,000 peptides [21].

### Global expression pattern

Although *H*. *tubifera* is regularly exposed to fluctuating temperatures in its shallow water habitat, different levels of thermal stress elicited observable changes in its gene expression profile. The expression profiles of sponges subjected to acute short-term thermal stress (34°C for 4 hours) showed higher correlation with controls maintained at 29°C while the transcriptome profiles of sponges subjected to longer thermal exposure (32°C for 12 hours and 34°C for 12 hours) were more similar to each other (Fig 1B). Although some sample replicates showed larger variability, principal component analysis was still able to differentiate the transcriptome profiles of sponges subjected to different temperature (Fig A in S1 File). Our results show that prolonged exposure to temperatures much higher than the average encountered by the sponge triggers substantial changes in gene expression that may influence cellular characteristics or behavior.

Differential expression analysis detected a total of 1,584 unique transcripts, referred to here as genes, exhibiting a significant change in expression across all treatments (Fig 1C). These results suggest that the sponge can rapidly deploy cellular mechanisms that support tolerance to increased temperatures. Most of the differentially expressed genes were up or downregulated by greater than four-fold relative to the controls (Table A in S1 File). The greatest number of differentially regulated genes were observed between the control (29°C) and samples heated at 34°C for 12 hours (1,010 genes). 294 genes remained differentially regulated in all samples subjected to elevated temperature while 178 differentially expressed genes were shared between samples exposed to 34°C for 4 hours and at 32°C for 12 hours. Only 32% (505 genes) of differentially expressed genes have homology to proteins in the UniProt database (Table B in S1 File). Of the genes with no UniProt matches, 644 exhibit similarity to sequences in the *A*. *queenslandica* reference genome (Table B in S1 File). Further studies are needed to elucidate the functions of these unannotated genes, which may include non-coding RNAs expressed from polyadenylated transcripts [22,23], as well as species-specific genes that are responsive to thermal stress.

By comparing the log2 fold change of genes that are commonly differentially expressed under the various treatments, we were able to determine the number that show a greater magnitude of change in expression under certain temperature regimes. Pairwise comparisons revealed that between the samples subjected to 34°C, more genes exhibited a greater magnitude of upregulation at 4 hours compared to 12 hours of exposure (142 vs. 104 genes), whereas more genes were downregulated in samples maintained at 34°C for 12 hours than for 4 hours (116 vs. 85 genes) (Fig 1D). Between samples exposed to different degrees of thermal stress, more genes were either up or downregulated in the samples exposed to 34°C for 4 or 12 hours relative to the samples maintained at 32°C for 12 hours (Fig 1E and 1F). This indicates that acute thermal stress at 34°C, regardless of duration, results in a greater change in gene expression levels compared to sublethal exposure at 32°C, which is still within the temperature range of the sponge habitat.

Downregulated sequences represent genes that are normally maintained at high levels in the sponge, which may include transcripts encoding proteins that form the first line of defense or transcripts with housekeeping functions that are generally energy intensive. Upregulated sequences are mostly stress-induced genes. Interestingly, thermal stress in *H*. *tubifera* resulted in more downregulation than upregulation of genes (Table A in S1 File). This finding is consistent with transcriptome-wide studies in other organisms, suggesting that widespread downregulation is a conserved phenomenon under stressful conditions [24–26]. This may be an adaptive mechanism that reflects the redirection of energy and resources towards the maintenance and repair of cellular machinery.

### Enriched functional groups

**Fig 2:**
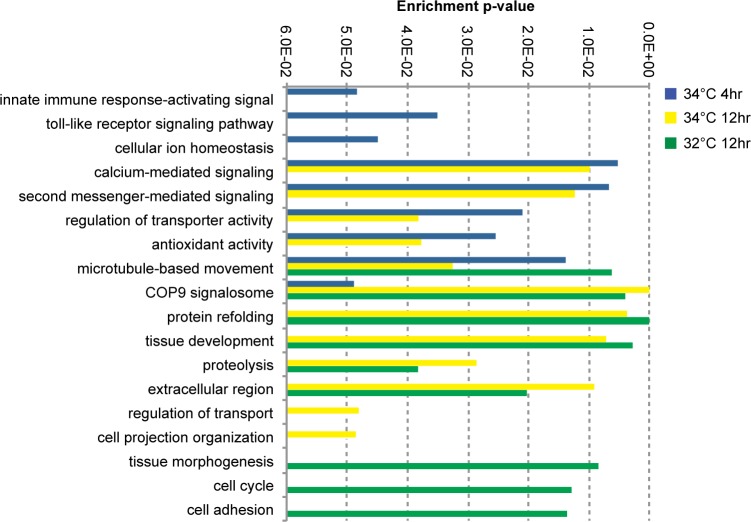
Gene ontology (GO) analysis for gene groups that are up or downregulated by greater than 4-fold under various temperature regimes. Enrichment p-values for selected terms are shown. Only GO terms with a p-value <0.05 (Fisher’s exact test) were considered significantly enriched.

Gene ontology (GO) enrichment analysis revealed that exposure of *H*. *tubifera* to elevated temperatures resulted in the differential expression of genes involved in the organismal stress response (Fig 2). Protective mechanisms that are enriched under acute short-term stress include antioxidant activity, toll-like receptor (TLR) signaling pathway, and innate immune response activation. Signaling mechanisms that are enriched include calcium-mediated signaling, cellular ion homeostasis, messenger-mediated pathways, transporter activity, and microtubule-based movement. This suggests that the sponge responds immediately to stress exposure by inducing signaling cascades that initiate various pathways involved in the cellular stress response and by producing transcripts that encode proteins that protect the cell from damage.

Longer exposure to elevated temperature (34°C for 12 hours) resulted in the differential expression of genes related to the constitutive photomorphogenesis 9 (COP9) signalosome *(CSN)*, protein refolding, tissue development, and proteolysis. The *CSN* is a protease complex with a key role in the DNA-damage response, cell-cycle control, apoptosis, and gene expression [27,28]. The activation of these functions indicate that sponge cells may have already sustained damage under these conditions.

Genes related to tissue morphogenesis, extracellular matrix, cell cycle and cell adhesion were enriched only in samples maintained at 32°C for 12 hours (Fig 2). Interestingly, stress response-related genes, such as glutathione transferase (*GST*) and the chaperones heat shock protein 90 (*Hsp90*) and *Bcl2*-Associated Athanogene 3 (*BAG3)*, were also upregulated at 32°C (Fig B in S1 File). This data suggests that exposure to the sublethal temperature of 32°C may be promoting tissue growth while, at the same time, activating the expression of protein folding chaperones that prepare the sponge for further encounters with environmental stressors. Effective acclimatization to sublethal temperature may increase the ability of the sponge to mitigate cellular damage upon exposure to a more extreme temperature.

Transcriptome-wide analysis in *H*. *tubifera* reveals insights into the mechanisms that are regulated in response to variable intensity and duration of thermal stress. This analysis showed that stress triggers a series of processes that function to maintain cellular homeostasis at all stages of stress exposure. Immediate responses, which are apparent in sponges subjected to short-term stress, involve modulating the oxidative response, immune response, and intracellular signaling. These responses are designed to prevent or minimize cellular damage at the onset of thermal stress. Under prolonged exposure, where the sponge may have already sustained some tissue damage, genes that are activated include those associated with protein refolding, tissue growth, cellular repair, and apoptosis. Induction of such functions may reflect sponge health, the ability to tolerate higher temperatures, and the potential for recovery and survival under continued stress conditions. It is interesting to note that exposure to sublethal temperature activates the expression of protein folding chaperones that provide sponge cells with some protection against extended or intensified stress conditions.

### Network responses to stress

**Fig 3:**
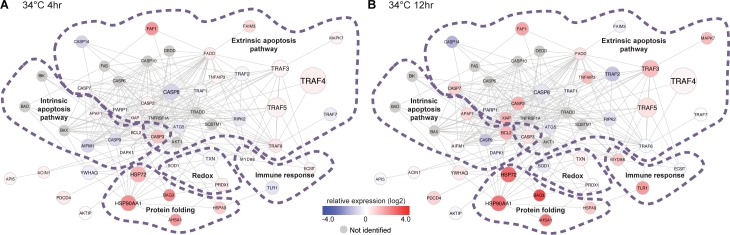
**Relative expression of sponge homologs of genes in the stress response-related protein network at (A) 4 hours and at (B) 12 hours of exposure to 34°C.** The network shown is based on the curated human protein interaction network. Relative expression is shown as the sum of the average fragments per kilobase per million (FPKM, log2 transformed) in each treatment relative to the control at 29°C for all genes with a best blastx hit matching the human gene in the network (blue, low; red, high; gray, no match in *H*. *tubifera*). Node size corresponds to the number of genes with the same UniProt annotation.

As is evident from the results of gene ontology enrichment, the cellular stress response involves the coordination of multiple pathways that are linked through protein-protein interactions. The sponge possesses homologs of many of the genes in these pathways but it is not known whether they also function in the same manner as their homologs in other organisms. Thus, to determine if these genes potentially retain similar functions in the sponge stress response, the sponge gene expression data was overlaid upon a protein interaction network based on well-curated annotations for human genes (Fig 3 and Table C in S1 File). Only the samples exposed to 34°C for 4 hours and 12 hours were included in this analysis as more robust responses were observed at this temperature than at the sublethal temperature of 32°C. This analysis enables the visualization of coordinated control or co-regulation, an indicator of conservation of function for gene homologs and of co-functionality for interacting genes [29–31]. In general, we observed that sponge homologs of genes within the stress response-related network, including genes that function in oxidative response, protein folding, immune response, and apoptosis, exhibit coordinated expression patterns that are consistent with known functions of their human homologs and that are indicative of their cooperative function in the sponge thermal stress response. Whether the sponge stress response-related interaction network also includes sponge-specific proteins different from those in the human network remains to be determined.

Not surprisingly, we found that the relative expression of heat shock protein 70 (*Hsp70)* and other protein folding genes was strongly upregulated in thermally stressed *H*. *tubifera* (Fig 3 and Fig B in S1 File). Hsp70 proteins are one of the most highly conserved groups of heat shock proteins [32]. They ensure the coordinated regulation of protein translocation processes, limit cellular damage by preventing aggregation of denatured proteins [33], and refold stress-denatured proteins [34]. We also found that *Hsp90* and ubiquitin were upregulated (Fig B in S1 File). This is in contrast to observations in adults of the Great Barrier Reef sponge, *R*. *odorabile*, where both *Hsp90* and ubiquitin expression were downregulated under thermal stress [9]. This difference in response may indicate that *R*. *odorabile* has greater sensitivity to thermal stress, as the decrease in expression may be due to widespread inhibition of gene transcription accompanying extensive cellular damage.

Exposure to thermal stress can induce the generation of reactive oxygen species, which can cause damage to tissues [35]. To counteract this, the organism produces genes that encode proteins with antioxidant activity. In *H*. *tubifera*, oxidative response genes such as thioredoxin (*TXN*), superoxide dismutase 1 (*SOD1*), and peroxiredoxin (*PRDX*) exhibited an increase in relative expression at higher temperatures (Fig 3 and Fig B in S1 File). Oxidative stress response genes have also been identified as differentially expressed in corals subjected to thermal stress [36,37]. The rapid response of antioxidant mechanisms in *H*. *tubifera* suggests that the sponge is able to cope with tissue damage that may be caused by oxidative stress associated with elevated temperatures.

Increased expression of heat shock proteins triggered by thermal stress can activate immune response-related genes, including toll-like receptors [38]. The toll-like receptor 1 (*TLR1*) and the mediator of TLR signaling, myeloid differentiation primary response gene 88 *(Myd88)*, exhibited increased relative expression under prolonged exposure to 34°C (Fig 3). TLRs initiate signal transduction pathways, which then induce the innate immune response and are important in the recognition of invading microbial pathogens [38]. These members of the innate immunity pathway, in turn, interact with genes in the intrinsic and extrinsic apoptosis pathways. Tumor necrosis factor alpha-induced protein 3 *(TNFAIP3)*, which serves as an inhibitor of TLR responses and apoptosis [39], was upregulated under stress. Similarly, the tumor necrosis factor receptor-associated factors (TRAFs), which act as adaptor proteins for a variety of receptors that regulate cell death and responses to stress [40], increased in expression upon exposure to 34°C. We also observed upregulation of the apoptotic protease activating factor 1 *(APAF1)*, which activates initiator caspases. Of the initiator caspases [41], only *CASP2* was upregulated, whereas *CASP8* and *CASP9* were downregulated. Initiator caspases cleave and activate executioner caspases, such as *CASP3* and *CASP7*, both of which were upregulated in the sponge. Executioner caspases degrade cellular components [41]. Induction of caspase-like activity was similarly observed during the early stages of thermal treatment in the sea anemone, *Anemonia viridis* [42]. Interestingly, inhibitors of apoptosis, such as X-linked inhibitor of apoptosis protein *(XIAP)* and B-cell lymphoma 2 *(Bcl2)*, as well as the anti-apoptotic *BAG3* chaperone, were upregulated upon exposure to 34°C at 12 hours. Despite the increase in expression of *APAF1* and executioner caspases, the downregulation of some initiator caspases, as well as the upregulation of known apoptosis inhibitors, suggests that the induction of cell death is tightly regulated in *H*. *tubifera* under stressful conditions.

### Thermal stress effects on regulatory pathways

**Fig 4:**
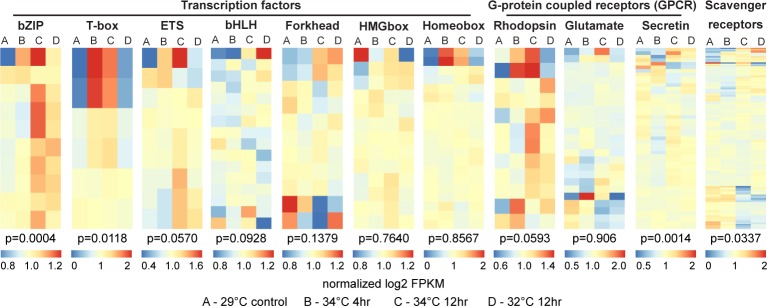
Relative expression of regulatory genes in *H*. *tubifera* during thermal stress exposure. Relative expression of genes encoding transcription factors (bZIP, T-box, ETS, homeobox, and forkhead), G-protein coupled receptors (rhodopsin, glutamate, and secretin GPCRs), and scavenger receptors. Relative expression was computed as the log2-transformed average FPKM value for each gene under each treatment normalized to the average of expression across treatments (blue, low; red, high). Each column represents one treatment (A, 29°C; B, 34°C, 4hr; C, 34°C, 12hr; D, 32°C, 12hr). Enrichment of genes under particular treatments was estimated using Fisher’s exact test (p-values shown).

Shifts in gene expression patterns can be attributed to changes in transcriptional activity. This is partly controlled by the abundance of different transcription factors that regulate the expression of specific genes in response to stimuli. We found that specific transcription factor groups exhibit distinct patterns of expression during thermal stress exposure, suggesting that these groups of factors play central roles in controlling the expression of genes commonly required to deal with the effects of increased temperatures. The most obvious trend observed for transcription factor families is an increase in expression at higher temperature with greater upregulation under prolonged exposure, as is evident for the bZIP, Tbox, ETS, bHLH, and forkhead families (Fig 4). In contrast, most HMGbox and homeobox family transcription factors were not responsive to thermal stimuli. While the specific gene targets of these transcription factor families remains to be determined, the increase in their expression during stress correlates with the upregulation of stress-related genes in the sponge. This suggests that while the responses observed upon acute exposure at 4 hours may be mainly due to rapid cellular responses involving mRNA turnover and protein translation or modification, longer term stress can influence global changes in the transcriptome through shifts in regulatory factor concentrations.

The interaction between an organism and its environment is mediated by transmembrane receptors. G-protein coupled receptors (GPCRs) and scavenger receptors form two of the largest gene families in sponges and are thought to provide the organism with a highly sophisticated repertoire of sensors to monitor its surroundings [20,43]. Upregulation of a subset of rhodopsin GPCRs at 12 hours of exposure at either 34°C or 32°C (Fig 4) suggests a link between thermal and light stress, which can be expected because intense solar irradiation typically correlates with increased sea surface temperatures. As such, the sponge may be able to trigger early protective responses in anticipation of seawater warming when it senses a rise in light intensity. Some specific glutamate GPCRs were upregulated upon exposure to 34°C. Glutamate GPCRs, such as the GABA-like and GRM-like receptors, may be involved in controlling the contraction of sponge canal system to regulate seawater filtration rate in response to environmental stimuli [44]. GABA receptors can either trigger canal contraction like in the demosponge, *Tethya wilhelma*, or they can be inhibitory, as in the freshwater sponge, *Ephydatia muelleri* [44,45]. Secretin GPCRs were downregulated at 4 hours of exposure to 34°C but increased in expression after 12 hours. These GPCRs are closely related to the adhesion GPCR family and their upregulation may be an adaptation that allows for better cell adhesion and the maintenance of sponge tissue integrity.

Changes in environmental conditions, such as a rise in temperature, may lead to the proliferation of opportunistic microorganisms and pathogens whose growth and virulence is favored by warmer temperatures [46,47]. In general, we found that scavenger receptors, which may be important for the discrimination between symbiotic and food bacteria [48], were downregulated under acute thermal stress (Fig 4). Animal lectins, which function in host defense by promoting aggregation of microbial cells for efficient phagocytosis [49], were also downregulated (Fig B in S1 File). Similarly, strong non-self recognition challenges, such as the selection of algal and bacterial endosymbionts, has been reported to cause a drop in the expression levels of lectins in corals during post-settlement [50]. In *H*. *tubifera*, changes in the population of cell surface receptors may serve to protect the sponge during thermal stress by aiding in the recognition and clearance of potential pathogens. However, despite the downregulation of these receptors, further exposure to stress and an increase in the abundance and virulence of pathogens may override the sponge immune system, eventually resulting in pathogen invasion. It is important to note that environmental perturbations that cause shifts in the sponge microbial assemblage or loss of groups of bacteria with critical metabolic functions will have a negative impact on the overall health of the sponge holobiont [17].

## Conclusions

Predicting the impact of climate change on important marine organisms, such as sponges, necessitates the elucidation of the cellular and molecular processes that contribute to their responses to elevated temperatures. This study represents the first transcriptome-wide survey of the sponge response to thermal stress. Using a high-throughput sequencing approach, we identified the potential mechanisms enabling *H*. *tubifera* to survive conditions of thermal stress and observed the different responses that are triggered at different stages of exposure. In *H*. *tubifera*, immediate stress response includes induction of heat shock proteins, antioxidants, and genes involved in signal transduction and innate immunity pathways while prolonged exposure to thermal stress affects expression patterns of genes involved in cellular damage repair, apoptosis, signaling and transcription. Differential deployment of a diverse repertoire of genes may allow the sponge to fine-tune its response to local conditions in the environment that they typically encounter. Thus, *H*. *tubifera*, which is normally located in shallow reef flats and is exposed to variable temperatures, may have a more robust response to temperature fluctuations compared to sponges found at deeper depths with colder and more stable temperatures. A similar phenomenon has been reported for several coral species, where individuals living in habitats that experience highly variable conditions exhibit greater expression of protective and metabolic genes [51,52].

For a more comprehensive understanding of sponge tolerance to climate change-related stressors, further comparative studies should be conducted on diverse species of marine sponges. The diversity within the sponge phylum and the relative plasticity of sponge cellular elements represent a treasure trove of gene innovations that may provide insights into the various pathways that have evolved to contribute to animal resilience [53]. It is likely that differences in ecological and physiological features of different sponges, and even their different life stages, will reflect variations in thermal tolerance and resilience that may have downstream effects on populations in areas where seawater temperatures begin to exceed tolerable temperatures. As sponges perform diverse roles in the marine ecosystem, changes in sponge distribution and abundance will have implications on reef functions. Thus, knowledge of gene expression responses in relation to organism physiology and health will support better assessment of sponge conservation status and contribute to the ability to predict reef trajectories under future climate conditions. Given the overwhelming evidence of coral decline due to climate-associated stressors, only those remarkably resistant taxa, such as sponges, are likely to survive the increasingly stressful marine environment.

## Materials and Methods

### Sample collection and thermal stress experiment

Sponges were collected by scuba diving in Malilnep Channel, Bolinao, Pangasinan (16.43530°N 119.94062°E) in September 2013 at depth of 1–3 meters. Collections were conducted with permission from the Philippines Department of Agriculture Bureau of Fisheries and Aquatic Resources (DA BFAR GP-0075-14). Sponge colonies were transported to the Bolinao Marine Laboratory outdoor seawater facility and were acclimated at 29°C, approximately the yearly average temperature at the reef site. Acclimation and thermal stress experiments were carried out in a shaded area receiving approximately 68 umoles/m^2^/s to avoid light stress. Thermal stress experiments were conducted in independently aerated 20 liter glass tanks (20.5 cm in depth) containing 5μm-filtered seawater with additional flow provided by 600 liter/hour submersible pumps. Experimental tanks were heated to 32°C or 34°C using 300 Watt submersible heaters. Water temperature in the tanks was monitored using underwater temperature loggers (HOBO PRO V2) set to record the temperature every 5 minutes. After a five-day acclimation period, sponges were exposed to thermal shock by immediate transfer to tanks with elevated temperatures (32°C and 34°C) or maintained at ambient temperature (29°C). One aquarium was used for each treatment. The sponges in the experiment represent two different individuals that were divided up into smaller pieces. One piece from each individual was included in every treatment. Tissues were collected after 12 hours for the control and all heated treatments, with an additional collection at 4 hours from 34°C. All tissues were stored in RNAlater (Ambion) and kept at 4°C for four hours then at -20°C for 12 hours. Samples were then transferred to liquid nitrogen for transport from Bolinao, Pangasinan to the Marine Science Institute, University of the Philippines, and stored at -80°C prior to molecular analyses.

### RNA extraction, quantity and quality assessment

Total RNA was extracted using Trizol Reagent (Invitrogen) following the manufacturer’s protocol with minor modifications, such as overnight precipitation at -80°C to obtain greater RNA yield. Contaminating genomic DNA was removed using the DNAfree kit (Ambion). Nucleic acid concentrations and ratios of absorbance at 260/280nm and 260/230nm were obtained using a BioSpec Nanodrop spectrophotometer (Shimadzu). The integrity of RNA samples was determined by electrophoresis on a native agarose gel with denaturing loading dye. RNA quality was further assessed using the mRNA Pico Series II assay on the Agilent Bioanalyzer 2100 System (Agilent Technologies) (Table D in S1 File).

### RNA sequencing and RNA quality control

Total RNA from duplicate samples from each experimental treatment were sent to Beijing Genomic Institute Tech Solutions (Hong Kong) Co., Limited. mRNA enrichment and preparation of barcoded cDNA libraries was done using the Illumina TruSeq RNA Sample Prep Kit protocol. Sequencing was conducted on the Illumina HiSeq 2000 platform with 100bp paired-end reads. After sequencing, the raw reads were filtered for adapter sequences and low-quality reads. Quality of the RNA-seq reads was visualized using FastQC 0.10.1 (Babraham Bioinformatics). Read quality control was performed using Trimmomatic 0.32 [54], which included discarding poor-quality bases (quality score below 3) at leading and trailing bases, scanning the read with a 4-base sliding window, cutting when the average quality per base drops below 30, dropping reads below 36 bases long, and trimming 18 bases from the start of the reads. Transcript abundance estimation was performed by mapping individual paired-end reads back to the *H*. *tubifera* non-redundant reference transcriptome assembly [21] using RNASeq by Expectation Maximization (RSEM) [55] with the Bowtie alignment method [56] included in the Trinity package suite [57]. Raw sequence reads were deposited in the NCBI Short Read Archive database (PRJNA274004). The reference transcriptome assembly is available on the Compagen site (compagen.org) [58]. This Transcriptome Shotgun Assembly project has been deposited at DDBJ/ENA/GenBank under the accession GFAV00000000. The version described in this paper is the first version, GFAV01000000.

### Differential gene expression analysis

Analysis of differentially expressed genes was conducted using duplicate samples from each experimental treatment. Analysis was done using the edgeR [59] package in R with expected counts obtained from transcript abundance estimation by RSEM. Expected counts from RSEM were converted to counts per million (CPM) and only genes with greater than 10 CPM in at least two samples were included in the analysis (S2 File). Genes that were up or downregulated by greater than 4-fold relative to the controls with an adjusted p-value <1x10^-5^ (Benjamini-Hochberg) were considered differentially expressed (S3 File). Control samples (29°C) from each species were compared to samples subjected to i) acute short-term stress (34°C at 4 hours), ii) acute long-term stress (34°C at 12 hours), and iii) intermediate long-term stress (32°C at 12 hours).

### Functional enrichment analysis

The *H*. *tubifera* transcriptome assembly was previously annotated by alignment to the UniProt database at an e-value cutoff of 1x10^-5^. The top blastx hit for each gene was used as input into Blast2GO [60] to retrieve gene ontology terms. Enrichment analysis for differentially expressed genes was performed using the topGO package in R [61]. Only the GO terms with a p-value <0.05 were considered significantly enriched.

### Protein network analysis

Protein-protein interactions for selected genes with known functions in the organismal stress response and that are represented by homologs in *H*. *tubifera* were retrieved from the STRING v10 functional protein interaction database [62]. Interactions were based on the well-curated human protein network. Relative expression of sponge gene homologs, computed as the sum of the average FPKM (log2 transformed) in each treatment relative to the control at 29°C, was overlaid on the interaction network and visualized in Cytoscape 3.1.1 [63]. FPKM values were obtained from RSEM output.

## Supporting Information

S1 File Combined supplementary information. (PDF)

S2 File Count matrix used for differential expression analysis. Transcript abundance estimation was performed by mapping paired-end reads back to the *H*. *tubifera* non-redundant reference transcriptome assembly using RSEM.
(XLSX)

S3 File Differentially expressed genes in *Haliclona tubifera* subjected to different temperature treatments. Analysis of differentially expressed genes was conducted with edgeR using expected counts obtained from the transcript abundance estimation. Only genes with greater than 10 counts per million in at least two samples were included in the analysis. Genes that were up or downregulated by greater than 4-fold relative to the controls with an adjusted p-value <1x10^-5^ (Benjamini-Hochberg) were considered differentially expressed.
(XLSX)
