# Supplementary material for: Gene Expression Dynamics Accompanying the Sponge Thermal Stress Response
Source: PLoS One. 2016 Oct 27;11(10):e0165368. doi: 10.1371/journal.pone.0165368 (PMC5082814; doi:10.1371/journal.pone.0165368)
Supplement: S1 File — (PDF) [file pone.0165368.s001.pdf]

Gene expression dynamics accompanying the sponge thermal stress response

Christine Guzman# and Cecilia Conaco*

Marine Science Institute, College of Science, University of the Philippines, Diliman, Quezon City, Philippines

#Current address: Okinawa Institute of Science and Technology Graduate University, 1919-1 Tancha, Onna-son, Okinawa 904-0495, Japan

*Corresponding author

Email: cconaco@msi.upd.edu.ph

S1 File. Combined supplementary information.

Figure A. Principal component analysis of normalized counts of *H. tubifera* genes from samples exposed to different temperature regimes. Only genes with counts per million (CPM) greater than 10 in at least 2 samples were included in the analysis. Replicates represent two independent sponge samples. The numbers in parentheses represent the proportion of variance explained by each principal component. PCA was computed in R.

Figure B. Relative expression of selected gene families in *Haliclona tubifera* during thermal stress exposure. Relative expression was computed as the log2-transformed average FPKM values for each gene under each condition normalized to the average of expression across treatments. Fisher's exact test was used to estimate enrichment of genes under specific treatments (p-values shown).

Table A. Number of differentially expressed genes detected in *Haliclona tubifera* subjected to thermal stress. Genes that were up or downregulated by greater than 4-fold relative to the controls with an adjusted p-value $<1x10^{-5}$ (Benjamini-Hochberg) were considered differentially expressed.

Table B. Number of differentially expressed genes detected in *Haliclona tubifera* subjected to thermal stress, that are unique or shared between different treatments. The number of genes with or without significant matches to the UniProt database or to *Amphimedon queenslandica* proteins at an evalue cutoff of $1x10^{-5}$ are shown.

Table C. Selected genes depicted in the stress response-related protein interaction network. Relative expression is based on the sum of the average fragments per kilobase per million (FPKM, log2 transformed) in each treatment relative to the control at 29°C for all genes annotated as belonging to each indicated gene family.

Table D. Collection details and quality assessment of RNA samples from various experimental treatments.

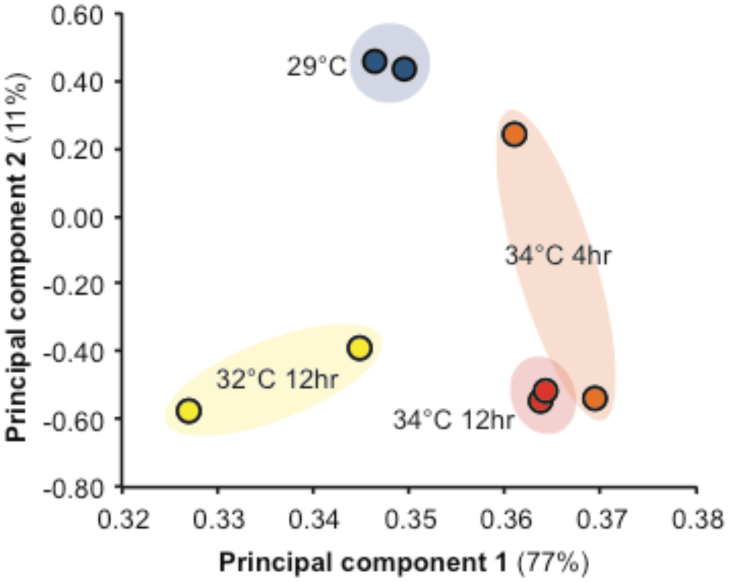

Figure A. Principal component analysis of normalized counts of *H. tubifera* genes from samples exposed to different temperature regimes. Only genes with counts per million (CPM) greater than 10 in at least 2 samples were included in the analysis. Replicates represent two independent sponge samples. The numbers in parentheses represent the proportion of variance explained by each principal component. PCA was computed in R.

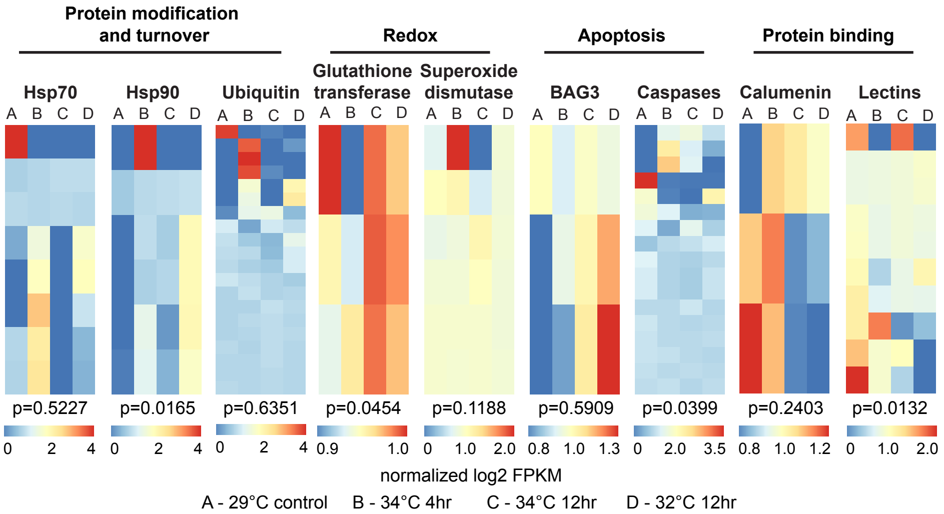

Figure B. Relative expression of selected gene families in *Haliclona tubifera* during thermal stress exposure. Relative expression was computed as the log2-transformed average FPKM values for each gene under each condition normalized to the average of expression across treatments (blue, low; red, high). Each column represents one treatment (A, 29°C; B, 34°C, 4hr; C, 34°C, 12hr; D, 32°C, 12hr). Fisher's exact test was used to estimate enrichment of genes under specific treatments (p-values shown).

Table A. Number of differentially expressed genes detected in *Haliclona tubifera* subjected to thermal stress. Genes that were up or downregulated by greater than 4-fold relative to the controls with an adjusted p-value <$1x10^{-5}$ (Benjamini-Hochberg) were considered differentially expressed.

	Number of DEGs	Number of up-regulated genes	UP Average fold change (log2)	Number of down-regulated genes	DOWN Average fold change (log2)	Range fold change (log2)
34°C 4hr	796	408	6.31	388	-4.90	-12.70 – 12.99
34°C 12hr	1010	478	5.70	532	-4.69	-13.50 – 13.58
32°C 12hr	892	381	4.98	511	-5.09	-14.47 – 14.53

Table B. Number of differentially expressed genes detected in *Haliclona tubifera* subjected to thermal stress, that are unique or shared between different treatments. The number of genes with or without significant matches to the UniProt database or to *Amphimedon queenslandica* proteins at an evalue cutoff of $1x10^{-5}$ are shown.

Treatments	Number of unique or shared DEGs	With UniProt hits	Without UniProt hits, but with *A. queenslandica* hits	No annotation
32°C 12hr	225	105	50	70
34°C 12hr	368	106	119	143
34°C 4hr	171	49	53	69
34°C 4hr, 34°C 12hr	153	46	46	61
34°C 4hr, 32°C 12hr	178	46	48	84
34°C 12hr, 32°C 12hr	195	69	49	77
34°C 4hr, 34°C 12hr, 32°C 12hr	294	84	70	140
Total	1584	505 (32%)	435 (27%)	644 (41%)

Table C. Selected genes depicted in the stress response-related protein interaction network. Relative expression is based on the sum of the average fragments per kilobase per million (FPKM, log2 transformed) in each treatment relative to the control at 29°C for all genes annotated as belonging to each indicated gene family.

Human gene name	Gene names of best blastx hit to *H. tubifera* genes	Relative expression at 34°C 4hr	Relative expression at 34°C 12hr	Number of genes
ACIN1	*ACINU*	0.254	0.133	1
AHSA1	*AHSA1*	1.768	2.156	1
AIFM1	*AIFM1/AIFM3*	-0.692	0.044	2
AKT1	-			
AKTIP	*AKTIP*	0.072	-0.091	2
APAF1	*APAF*	0.189	0.612	1
API5	*API5*	0.182	-0.055	1
ATG5	*ATG5*	-0.353	-0.434	1
BAD	-			
BAG3	*BAGS*	2.042	2.835	1
BAX	-			
BCL2	*B2CL1*	0.177	1.139	2
BIK	-			
CASP10	-			
CASP14	*CASPE*	-0.759	-1.164	1
CASP2	*CASP2*	0.229	1.222	3
CASP3	*CASP3*	1.060	0.474	3
CASP6	-			
CASP7	*CASP7*	0.086	0.707	4
CASP8	*CASP8*	-0.755	-0.370	13
CASP9	*CASP9*	-0.631	-0.904	1
DAPK1	*DAPK1*	-0.037	-0.242	5
DEDD	-			
ECSIT	*ECSIT*	0.169	-0.120	1
FADD	*FADD*	0.350	0.440	1
FAF1	*FAF1*	1.782	1.351	3
FAIM3	*FAIM1*	0.583	-0.174	1
FAS	-			
HSP72	*HSP70/HSP7C/HS71L/HS71A*	1.315	2.251	11
HSP90AA1	*HSP90A/HSP90B*	1.984	2.174	11
HSPA9	*GRP75*	0.904	0.778	1
MAPK7	*MP2K7*	0.191	1.030	1
MYD88	*MYD88*	0.056	0.820	1
PARP1	*PARP1/PARP2/PARP3/PARP4/PARP6*	-0.075	-0.175	8
PDCD4	*PDCD4/PDCD6*	0.370	0.655	4
PRDX1	*PRDX4/PRDX5/PR*	0.318	0.099	5

	DX6			
RIPK2	*RIPK4/DUSTY*	-0.353	-0.434	2
SOD1	*SODC*	0.075	-0.300	3
SQSTM1	-			
TLR1	*TLR1*	-0.457	1.508	4
TNFAIP3	*TNAP3*	0.191	1.030	1
TNFRSF1A	-			
TRADD	-			
TRAF1	*TRAF1*	-0.148	-0.147	3
TRAF2	*TRAF2*	-0.163	-0.912	8
TRAF3	*TRAF3*	0.177	0.969	20
TRAF4	*TRAF4*	0.264	0.146	65
TRAF5	*TRAF5*	0.224	0.424	28
TRAF6	*TRAF6*	0.399	-0.051	2
TRAF7	*TRAF7*	-0.283	0.026	5
TXN	*THIO*	-0.127	0.244	6
XIAP	*XIAP*	0.236	1.117	1
YWHAQ	*1433Z/1433B/1433G/143GB*	-0.261	0.311	9

Table D. Collection details and quality assessment of RNA samples from various experimental treatments.

Species	Date of collection; Locality	Treatment	RNA integrity number (RIN)	28S:18S
Haliclona tubifera	September 2016; Malilnep Channel, Bolinao, Pangasinan, Philippines	29°C 4hr	8.7	1.20
		29°C 4hr	8.8	1.30
		34°C 4hr	8.4	1.40
		34°C 4hr	8.7	1.30
		34°C 12hr	8.4	2.10
		34°C 12hr	8.5	1.40
		32°C 12hr	9.3	1.90
		32°C 12hr	9.1	1.50
